# Soil nitrous oxide flux following land‐use reversion from Miscanthus and SRC willow to perennial ryegrass

**DOI:** 10.1111/gcbb.12541

**Published:** 2018-08-30

**Authors:** Jon P. McCalmont, Rebecca Rowe, Dafydd Elias, Jeanette Whitaker, Niall P. McNamara, Iain S. Donnison

**Affiliations:** ^1^ Institute of Biological, Environmental and Rural Sciences (IBERS) Aberystwyth University Gogerddan Aberystwyth Wales, UK; ^2^ Centre for Ecology and Hydrology Lancaster Environment Centre Lancaster UK

**Keywords:** crop reversion, energy crops, greenhouse gas balance, land‐use change, Miscanthus, N_2_O, nitrous oxide, Nutrients, perennial ryegrass, Willow

## Abstract

Decarbonization of the world's energy supply is essential to meet the targets of the 2016 Paris climate change agreement. One promising opportunity is the utilization of second generation, low input bioenergy crops such as Miscanthus and Short Rotation Coppice (SRC) willow. Research has previously been carried out on the greenhouse gas (GHG) balance of growing these feedstocks and land‐use changes involved in converting conventional cropland to their production; however, there is almost no body of work understanding the costs associated with their end of life transitions back to conventional crops. It is likely that it is during crop interventions and land‐use transitions that significant GHG fluxes might occur. Therefore, in this study, we investigated soil GHG fluxes over 82 weeks during transition from Miscanthus and SRC willow into perennial ryegrass in west Wales, UK. This study captured soil GHG fluxes at a weekly time step, alongside monthly changes in soil nitrogen and labile carbon and reports the results of regression modelling of suspected drivers. Methane fluxes were typically trivial; however, nitrous oxide (N_2_O) fluxes were notably affected, reverted plots produced significantly more N_2_O than retained controls and Miscanthus produced significantly higher fluxes overall than willow plots. N_2_O costs of reversion appeared to be contained within the first year of reversion when the Miscanthus plots produced an average pregrass flux of 0.13 mg N_2_O m^−2^ hr^−1^ while for willow, this was 0.03 mg N_2_O m^−2^ hr^−1^. Total N_2_O emission from reversion increased the carbon cost over the lifetime of the Miscanthus from 6.50 to 9.91 Mg CO_2_ eq. ha^−1^ while for the willow, this increase was from 9.61 to 10.42 Mg CO_2_ eq. ha^−1^. Despite these significant increases, the carbon cost of energy contained in these perennial crops remained far lower than the equivalent carbon cost of energy in coal.

## Abstract 2

There are likely to be greenhouse gas peak “hotspots” during land‐use change and agronomic interventions. This study investigated soil N_2_O flux at the end of crop life for two energy crops, Miscanthus and willow, during land‐use reversion to grassland (the previous land‐use). This is the first time in the literature that side‐by‐side energy crops and reversion systems have been compared and followed at a weekly resolution over 2 years. Mean and peak fluxes are quantified along with an indication of the time taken for these increased levels of emissions to return to a reasonable ambient for that particular land‐use.

## 1 INTRODUCTION

There is a need to decarbonize energy production if we are to reduce the negative impacts of anthropogenic climate change (IPCC 2014) and meet the ambitions of the 2016 Paris agreement (UNFCC 2015). Energy production from renewables, such as solar, wind and biomass, already plays a significant role in the energy mix. In 2016, the European Union produced 17% of its total primary energy from renewable sources with bioenergy supplying 53% of this (ec.europa.eu, 2017). For the United Kingdom, in 2015, renewable electricity represented 24.6% with biomass supplying 70.7% of this (DUKES 2016).

While bioenergy can help to mitigate fossil fuel carbon emissions, there is a need to understand the lifecycle emissions of all greenhouse gases (GHGs) associated with bioenergy production, including methane (CH_4_) and nitrous oxide (N_2_O). Due to their high global warming potentials (GWP), these trace gases can play a pivotal role in the GHG budgets of biomass feedstocks and fluxes from soil are a key source (Behnke, David, & Voigt, 2012; Palmer, Forrester, Rothstein, & Mladenhoff, 2014; Roth, Finnan, Jones, Burke, & Williams, 2015; Roth, Jones, Burke, & Williams, 2013).

Methane emissions are typically not significant from temperate agricultural soils (Snyder, Bruulsema, Jensen, & Fixen, 2009), however, soil N_2_O emissions from agriculture are the primary source of global atmospheric N_2_O, particularly from fertilised systems (Dobbie, McTaggart, & Smith, 1999; Reay et al., 2012). Significant progress has been made in quantifying growing cycle N_2_O emissions from biomass production but the start and end of crop life are still poorly quantified, despite these being the periods of greatest soil perturbation and C and N inputs (Whitaker et al., 2017). Although there have been some studies investigating GHG fluxes during conversion periods to energy crops (e.g., Nikièma, Rothstein, & Miller, 2012; Oates et al., 2015; Palmer et al., 2014; Roth et al., 2013; Saha et al., 2017) and others investigating the impacts of fertiliser additions on N_2_O emissions (Behnke et al., 2012; Drewer, Finch, Lloyd, Baggs, & Skiba, 2012; Duran, Duncan, Oates, Kucharik, & Jackson, 2016; Gauder, Butterbach‐Bahl, Graeff‐Honninger, Claupein, & Wiegel, 2012; Hellebrand, Scholz, Kern, & Kavdir, 2005; Jørgensen, Jorgensen, Nielsen, Maag, & Lind, 1997; Roth et al., 2015; Ruan, Bhardwaj, Hamilton, & Robertson, 2016), surprisingly, little work has considered GHG dynamics at the end of cropping cycles with reversion back to more typical agricultural systems. One study looked into the reversion of a 20‐year‐old *Miscanthus* x *giganteus* plantation into wheat production and set‐aside (Drewer, Dufossé, Skiba, & Gabrielle, 2014; Dufossé, Drewer, Gabrielle, & Drouet, 2014). This study concluded that cumulative sums for the year following reversion of Miscanthus had increased by up to 200%. Unfortunately, although, peak flux rates, seasonal trends and timings of emissions were not reported. Another study (Pacaldo et al., 2014) investigated the removal of willow and compared to harvesting/regrowth cycles, but results were limited to carbon flux and biomass pools and did not consider N_2_O.

Nitrous oxide emissions from soils are primarily driven by changes in nutrient and oxygen status (Zechmeister‐Boltenstern, Schaufler, & Kitzler, 2007), typically by soil biota either nitrifying available ammonium (NH_4_^+^) to nitrate (NO_3_^−^) or supplementing a lack of available oxygen by utilising oxygen atoms from soil NO_3_^−^ in denitrification (Butterbach‐Bahl, Baggs, Dannenmann, Kiese, & Zechmeister‐Boltenstern, 2013). While this limitation in soil oxygen is generally understood to be the result of waterlogging, it can also be caused by oxygen utilization and depletion through high concentrations of respired CO_2_. Soil organic matter decomposition, with interactions between soil carbon, water‐filled pore space (WFPS) and nutrients show strong correlations with N_2_O production (Weier, Doran, Power, & Walters, 1993), therefore, changes in land‐use that impact water (changes in soil bulk density, vegetative cover or crop utilization of soil water) or nutrient/labile carbon status may have direct impacts on soil N_2_O fluxes. Conversion into, and reversion from, perennial bioenergy crops is likely to result in perturbation of all these parameters through additions of nitrogen and carbon in crop and root residues and changes in water (and therefore oxygen) status through cultivation and crop operations (Zechmeister‐Boltenstern et al., 2007). It might be expected that there would be “hotspot” periods of peak fluxes at times of increased soil disturbance, crop residue additions, fertilisation events, etc. and, in perennial systems, these might be expected to be greatest during land‐use changes between crops and systems.

In this study, we report the findings of an 82‐week reversion experiment from two perennial energy crops, *Miscanthus* × *giganteus* and *Salix viminalis*, back into grassland of *Lolium perenne* (the previous land‐use) where N_2_O fluxes were sampled weekly along with biomass assessments, biomass carbon/nitrogen ratios and nutrient dynamics. We hypothesized that reversion from these bioenergy crops into more conventional agriculture would result in increased N_2_O fluxes during the transition period and aimed to capture the magnitude and timing of this and investigate potential drivers. This study is unique in following N_2_O fluxes at high frequency over an entire transition period for two adjacent energy crop reversions to conventional agriculture alongside retained control plots.

## 2 MATERIALS AND METHODS

### 2.1 Site description

The experimental site was located near Aberystwyth, Wales, UK (52°24′49″N 4°2′35″W). The dominant soil type is an imperfectly drained Dystric Cambisol (FAO, World Harmonized Soil Database), with a silty clay loam soil texture and a pH range of 5.6–5.9 across the experimental area. The site was originally established into pre‐existing long‐term agricultural grassland as a replicated block trial of bioenergy crop yield traits in 2009 (Purdy et al., 2015), no fertiliser was applied to either crop during the lifetime of the project. For this study, two commercial bioenergy crop genotypes, *Miscanthus* × *giganteus* (hereafter Miscanthus) and *Salix viminalis* (genotype Tora, hereafter willow), were selected for reversion to *Lolium perenne* (perennial ryegrass). Making use of the previous experimental design of four replicates per genotype, two replicate plots of each crop were reverted and two were retained. Plots were selected for reversion by random number allocation. The Miscanthus trial plots measured 8.5 m × 7.2 m (61.2 m^2^) and had been planted at a density of 2 plants/m^2^. The willow plots were the same width (8.5 m) but were 26.4 m long and planted at a density of 1.6 plants/m^2^, for the purposes of this reversion experiment, an equivalent area to the Miscanthus plots was utilised.

### 2.2 Miscanthus reversion (Miscanthus—forage kale—ryegrass)

Based on commercial practice for Miscanthus removal, the plants in the reversion plots were allowed to regrow for 2 months following harvest until glyphosate herbicide application on 23 June 2015 (5 L/ha, Roundup Biactive, Monsanto UK, Cambridge, UK). After allowing 5 weeks for effective herbicide uptake, the aboveground biomass was cut and removed (27 July 2015) and the soil cultivated by rotovator and roller to produce a seedbed. As might be typical in commercial practice, a catch crop of forage kale (*Brassica oleracae*, Caledonia) was planted (7 August 2015) to utilise the residual nutrients of the decomposing rhizomes and to provide an overwinter cover. The forage kale was harvested in early February 2016 and the reversion plots left fallow until 3 June 2016 when they were rotovated, rolled and seeded with perennial ryegrass. The ryegrass was subsequently fertilised with ammonium nitrate fertiliser (34.5% N, Nitram, CF Fertilisers, Cheshire, UK) on the 14 July 2016 at the equivalent of 250 kg/ha.

### 2.3 Willow reversion (Willow—fallow—ryegrass)

Willow reversion plots were cut back to ground level on the 20 May 2015 and allowed to regrow for 1 month to provide sufficient leaf growth to facilitate uptake of herbicide. Plots were then sprayed with glyphosate at the same time and rate as the Miscanthus. Unlike the Miscanthus, the willow reversion plots were then left fallow until 24 May 2016 allowing the stumps to begin to decay. A stump grinder was then used to break up the willow stools with all material retained within the plots. From this point on, the Miscanthus and willow reversion plots followed identical cultivation, perennial ryegrass seeding and fertilisation treatment with operations carried out on the same days and at the same rates. For both crops, plant kill was absolute with no volunteer seedlings or new shoot growth observed following herbicide applications. The retained willow plots were not cut back as all plots had been routinely harvested in February 2014 and were following a two‐year harvest cycle.

### 2.4 Prereversion baseline biomass sampling

Belowground biomass (BGB) was sampled prior to reversion (19 June 2015) from both crops to give an indication of biomass dry weight and composition that would be added to the soil organic pool during the reversion process. Two randomly selected individual plant rhizomes and associated coarse roots were removed from each reversion plot. Soil monoliths (centred on each plant) 0.5 m^2^ and 30 cm deep were removed, capturing the entire rhizome for each plant and representing the area each plant would occupy in a 2 plant/m^2^ planting density. These were washed through a 2 mm sieve to remove mud and stones, and dried at 40°C until constant weight. Roots and rhizomes were then separated by hand, weighed and scaled by planting density to give total BGB per unit area. While it is reasonable to assume that the entire rhizome for an individual plant had been recovered, this would not be the case for the root systems. Therefore, as only coarse roots were retrieved in this process, estimates of biomass added to the belowground carbon and nitrogen pools should be considered a minimum with the assumption that there would be more root biomass than has been captured and reported here.

Following Miscanthus removal, the forage kale transition crop in these reversion plots added further biomass to the belowground pool. This was assessed (10 December 2015) from within a single 4 m^2^ quadrat located at the centre of each of the reversion plots. All kale plants within these quadrats were collected by hand pulling from the soil. Roots were separated from the aboveground biomass and dried and weighed as with the energy crops. Again, results should be considered a minimum root mass although in the case of this crop, the distinct taproot structure and limited root development from 5 months’ growth of an annual plant would suggest that a greater proportion of the root biomass was recovered.

Similarly to the Miscanthus, two willow stools were excavated from each willow reversion plot and washed, dried to constant weight and the roots and stools separated. As with the Miscanthus, given the difficulty in recovering willow root biomass under field conditions, only stool biomass could be considered reliable while root biomass should be considered as the minimum biomass that would have been added as soil organic matter.

### 2.5 Carbon and Nitrogen in belowground biomass

Two subsamples of the dried belowground biomass (Miscanthus rhizome/roots, willow stool/roots and kale root) were taken and ground to <2 mm using a Retsch mill (SM100, Retsch, Haan, Germany) before being further milled to a fine, homogeneous powder using cryogenic milling in liquid nitrogen (6870 Cryo‐mill, SPEX, Stanhope, UK). Samples were analysed for total C and N concentrations using a LECO Truspec elemental analyser (TruSpec CN analyser, Leco Corp., St. Joseph, MI, USA).

### 2.6 Crop harvest yield assessments

Annual harvest yield assessments followed the standard protocols which have been in operation since site establishment in 2009. For both Miscanthus and willow, harvest takes place during the dormant period in late winter/early spring, between January and March.

Typical for Miscanthus cultivation, the first year's growth was minimal and not considered to be commercially relevant. From the second year (2010) onward, a quadrat was marked out within each plot capturing 12 plants in total (4 × 3), leaving one row along the edges of the plot to act as a buffer. All plants within the harvest quadrat were cut and chipped in bulk to produce a mean weight per plant over 12 plants. Homogenised subsamples (300–500 g) were collected from the bulk and dried to constant weight to determine moisture content. Estimates of yield per area were calculated through multiplying by the planting density. The rest of the plots were then cut back with all material removed.

Harvest yield was assessed for the willow every 2 years, beginning in 2012; this followed cutting back of the first year's growth at the end of 2009 to encourage stool development to maximise stem density. Two predetermined rows of plants across each replicate plot were designated as the yield sampling quadrat. All plants within these two rows (totalling 26 plants) were cut, chipped and weighed in bulk to determine a mean yield per plant with a homogenised subsample of 300–500 g taken to assess moisture content. Following the yield subsample assessments, the rest of the plots were cut back with all biomass removed from the site. As with the Miscanthus, mass per plant was multiplied by planting density to estimate yield per area. This process was repeated as a 2‐year harvest in 2014 and again in 2016.

### 2.7 N_2_O flux measurements

Fluxes of N_2_O were measured weekly for 82 weeks from 2 June 2015 to 31 January 2017 using the static chamber method. Two permanent collars were inserted into the soil in each plot of each treatment at ~2–3 cm depth to minimise fine root and mycorrhizal disturbance (Heinemeyer et al., 2011; Mills, Glanville, McGovern, Emmett, & Jones, 2011). These collars remained in place throughout the study period, only being removed briefly during cultivation operations. The internal volume of each collar was calculated from the mean of three internal height measurements after insertion (from the soil surface inside to the lip of the collar) and added to the known volume of the chamber lids (0.0251 m^3^) for gas concentration calculations. At each sampling, chamber lids were placed onto the collars and sealed with spring clamps before sampling. Headspace gas samples (10 ml, 0.033% of total chamber headspace volume) were taken through a butyl rubber septum using the static chamber method (Baker et al., 2003; Parkin & Venterea, 2010) at 0, 15, 30 and 45 min (postenclosure) and injected into 3 ml gas‐tight borosilicate glass vials (Labco, Lampeter, UK). Analysis of these samples was carried out by gas chromatography (PerkinElmer Autosystem XL Gas Chromatograph, PerkinElmer, Waltham, MA, USA). Each chamber covered an area of 0.13 m^2^; with two chambers in each 61.2 m^2^ plot, and this represented chamber coverage of 0.42% of the total plot area. Temperature loggers (Hobo pendant logger, ONSET, Bourne, USA) were placed inside the chambers throughout each sampling period to record internal air temperatures at one‐min intervals; the mean of this during chamber closure was combined with the concentration gradients over time to produce an estimate of gas flux using the *Flux* package (Jurasinski, Koebsch, Guenther, & Beetz, 2014) in R (vers. 3.2.0, R Core Team 2015). For this work, as in Dufossé et al. (2014) and others, the CO_2_ flux, which typically exhibits a reliable flux gradient, was used as a quality control indicator for the main focus, the N_2_O flux. In total, nine of 1313 individual chamber fluxes were rejected due to having CO_2_ flux gradients with an *R* ^2^ < 0.9. Static chamber sampling ran from the 2 June 2015 to the 31 January 2017. In total, there were 82 rounds of sampling with two chambers sampled per plot across the two treatments and controls.

### 2.8 Ancillary measurements

At each GHG sampling event, soil temperature was measured at the centre of each plot using a calibrated 10‐cm‐long stab probe (Testo 104, Testo Ltd. Hampshire, UK). At the same location, three soil volumetric moisture measurements were made (ML3 soil moisture probe, Delta‐T Devices, Cambridge, UK) with the mean value per plot being used for analyses. Additional meteorological data (precipitation, air temperature, solar radiation) were available from a nearby (2 km distance) automatic weather station (Campbell Scientific, Utah, USA; McCalmont, McNamara, Donnison, Farrar, and Clifton‐Brown (2015)).

Soil samples (0–15 cm) were also taken following cultivation and reversion to determine bulk density. One sample was taken per plot using a 4.7 cm wide noncompressive, split‐tube soil auger (Eijkelkamp, Giesbeek, the Netherlands). Soil cores were oven‐dried to constant weight, and sieved to <2 mm, mass of these known volumes was used to calculate soil bulk density (g/cm^3^). The volumetric moisture measurements and soil bulk densities were combined to calculate soil water‐filled pore space (WFPS) for each gas flux sampling following Elliott, Heil, Kelly, and Monger (1999). Soil bulk density was also used to convert mass units (e.g., g N (kg soil)^−1^) to area estimates (i.e., kg N ha^−1^).

### 2.9 Soil nitrogen and labile carbon availability

In addition to the bulk density sampling, 0–15 cm soil cores were taken using the auger from a 2 m radius around the static chamber collars at monthly intervals from 21 June 2015 to 4 January 2017. Soils were analysed for inorganic nitrogen (N_min_) and dissolved organic carbon (DOC) with two soil cores per plot (one per chamber). Analysis was carried out on 5 g subsamples of fresh soil using a 1 M potassium chloride (KCl) extraction for NH_4_^+^ and NO_3_^−^ and with pure water for extraction of DOC. The KCl extractions were analysed through colorimetric segmented flow analysis (SFA) (AA3, Seal Analytical, Southampton, UK). DOC analysis was carried out using catalytically‐aided combustion/nondispersive infrared detection (NDIR) (Shimadzu TOC‐L_CNP_ with TNM‐L, Kyoto, Japan).

### 2.10 Data analysis

#### 2.10.1 Differences in N_2_O flux between land‐use treatments

Comparisons of N_2_O flux were compared between land‐use treatments and between crops using linear mixed effects models (Laird & Ware, 1982) within the *nlme* package in R (Pinheiro, Bates, DebRoy, & Sarkar, 2015). To provide this comparative data set for testing between treatments, cumulative fluxes over the sample period were calculated for each chamber by simple summing of values. Shapiro–Wilk tests were used to assess normality of the summed data set and, where necessary, log transformations were applied. A constant of 1 was added to all summed values to prevent log transformation from converting values lower than 1 to a negative value. To investigate the persistence of any impacts found on N_2_O flux into the subsequent grass crop following the individual reversion treatments, two time periods were considered, pre‐ and postgrass sowing.

The linear mixed effects model considered impacts on summed fluxes with crop (Miscanthus and willow) and treatment (reverted or retained) as fixed factors, and plot included as a random factor to account for nonindependence due to repeated measures. Model residuals were graphically checked for normality using Q–Q plots and histograms. Likelihood Ratio Tests were performed to determine the significance of individual parameters in the model. Marginal R^2^ values were calculated for models using the r.squaredGLMM function in the R package MuMin (Barton, 2017).

#### 2.10.2 Belowground biomass and C/N additions to belowground soil pools

Statistical comparisons of belowground biomass and C and N percentage for roots and rhizome/stools were carried out using ANOVA with Tukey HSD for multiple comparisons of means. Specific C and N percentages for biomass components (rhizome/stool and roots) were combined with biomass assessments to estimate potential additions to soil pools following reversion and cultivation.

#### 2.10.3 Regression modelling for potential drivers

To investigate the magnitude and significance of the potential drivers of N_2_O production, multiple regression was performed for each land‐use. A positive constant was first applied to the flux data which were then transformed using the Box‐Cox power transformation (Moulin et al., 2014) from the CAR package in R (Fox & Weisberg, 2011) and tested for normality graphically and using the Shapiro–Wilk test. Corresponding mean values for soil moisture and air and soil temperature were calculated for the same time points and added to the dataset. Nutrient data were only measured at monthly intervals so, following similar calculation of means by land‐use treatment, these data were linearly interpolated at a daily time step with values then matched to the corresponding dates for the weekly flux measurements. Stepwise, best fit parameter substitution, both forward and backward, was used to derive models with the lowest AIC (Akaike's Information Criteria) score from the resulting parameters as follows:

*T*_a_, Air temperature
*T*_s_, Soil temperature
WFPS, Water‐filled pore space
NO_3_, Nitrate at 0–15 cm soil depth
NH_4_, Ammonium at 0–15 cm soil depth
NH_4_/NO_3_, Ammonium/nitrate ratio
DOC, Soluble organic carbon

Both individual correlations and all pairwise interactions were tested for each of the experimental treatments. Parameters found to be adding significant information to the model were then tested to determine their relative contributions (%) to the total variance explained by it, following methods set out in Lindeman, Merenda, and Gold (1980) and Kruskal (1987) and implemented using the R package *relaimpo* (Grömping, 2006). The calculated percentage contributions to the total explanation of variance in each model for each significant parameter were bootstrapped by recalculating 100 times to produce 95% confidence intervals. (±figures throughout represent the Standard Error of the Mean)

#### 2.10.4 GHG balance implications of N_2_O reversion fluxes

To provide some context for the global warming potential (GWP) of these reversion “hotspot” fluxes, we consider the total sum of N_2_O (in terms of CO_2_ equivalent) emitted to the atmosphere during the pregrass reversion period and relate it to the GWP costs of producing the biomass over the lifetime of the crop. Mean hourly fluxes (mg N_2_O m^−2^ hr^−1^) recorded within the pregrass period were multiplied by 24 to produce a daily total, unit converted to Mg/ha and then linearly interpolated at a daily time step between the 47 weekly values and summed to a total flux. Results were then multiplied by 298 (IPCC 2007) to give GWP as CO_2_‐eq.

These results were then considered in the context of life cycle assessments (LCA) of the overall carbon intensity of producing these harvest yields over time in CO_2_ equivalent emissions per MJ of energy in dry matter (CO_2_ eq. MJ^−1^). Energy contents were assumed for Miscanthus biomass at 17.95 GJ Mg^−1^ DM (Felten, Froba, Fries, & Emmerling, 2013) and for willow at 19.8 GJ Mg^−1^ DM (Heller, Keoleian, Mann, & Volk, 2004). The GHG cost of producing this energy for Miscanthus is assumed at 4.40 g CO_2_ eq. MJ^−1^ (Hastings et al., 2017), this value incorporates the entire supply chain, from rhizome propagation, harvest costs, biomass chipping and pelletization and transport (100 km) to the furnace. For willow, a figure of 9.16 g CO^2^ eq. MJ^−1^ was calculated by the same authors following the same methodology (Hastings, unpublished data, pers. comm.), the higher willow figure reflecting a much higher moisture content at harvest with associated increased transport and drying costs. These figures were then contrasted to the carbon intensity of a relevant fossil fuel substitute, in this case coal, with a carbon intensity of 120.89 g CO_2_ eq. MJ^−1^ (Hastings et al., 2009). The carbon intensity value includes exploration, recovery, processing and transportation of the coal (100 km) to the furnace.

## 3 RESULTS

### 3.1 Nitrous oxide fluxes

**Figure 1:**
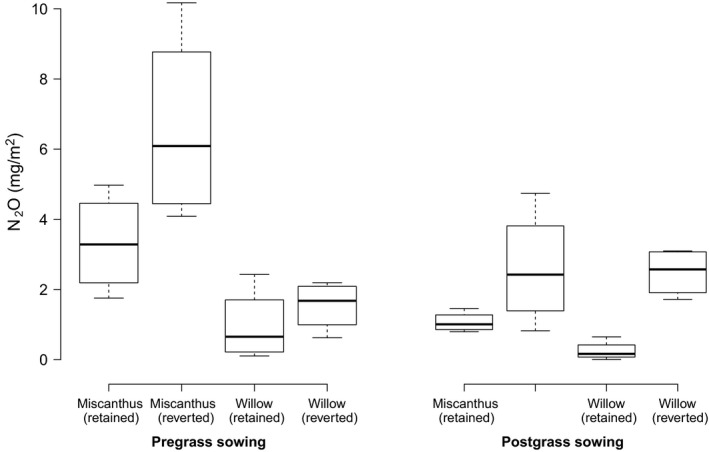
Boxplots of the sum of N_2_O fluxes (measured at individual static chambers) compared between retained controls and reversion treatments. Two periods are compared; pregrass sowing (left) which covers the period from the day after spraying out of the existing crop to cultivation for the new grass crop and postgrass sowing (right), from the period following cultivation and grass sowing to the end of the study. Solid bars represent the median values, whiskers represent the minimum and maximum values from each chamber with no values exceeding the 1st or 3rd quartile ±1.5 times the interquartile range

Comparison of the sums of all N_2_O fluxes measured prior to the cultivation and sowing with ryegrass (pregrass) demonstrated that N_2_O emissions had increased significantly following reversion of both willow and Miscanthus (LRT = 6.56, *p* = 0.01). Interactions between crop and treatment were not found to be significant (LRT = 0.6, *p* = 0.44) showing that both soils responded to reversion in the same way. Crop type was significant with lower average fluxes in the willow plots than in the Miscanthus (LRT = 18.9, *p* = <0.0001) for both retained and reverted treatments. Marginal R^2^ value for the full model was 0.73 with treatment accounting for more of the variance (*R* ^2^_m_ = 0.60) than crop (*R* ^2^_m_ = 0.13). Figure 1 shows boxplots for both pre‐ and postgrass sampling comparing both crop and treatment. There was a significant difference between pre‐ and postgrass flux sums (LRT = 22.6, *p* < 0.0001) but this was not consistent between crops. For the reverted Miscanthus, fluxes were lower postgrass (mean 0.08 ± 0.01 mg N_2_O m^−2^ hr^−1^) compared to pregrass (0.13 ± 0.02 mg N_2_O m^−2^ hr^−1^), for reverted willow the opposite was true (postgrass 0.08 ± 0.01 mg N_2_O m^−2^ hr^−1^, pregrass 0.03 ± 0.01 mg N_2_O m^−2^ hr^−1^). In both crop types reverted plots continued to show higher fluxes overall then retained controls (LRT = 16.1, *p* = <0.0001) but, unlike in the pregrass period, postgrass there was no significant difference in flux magnitude between the two reverted crop types (LRT = 2.6, *p* = 0.12).

**Figure 2:**
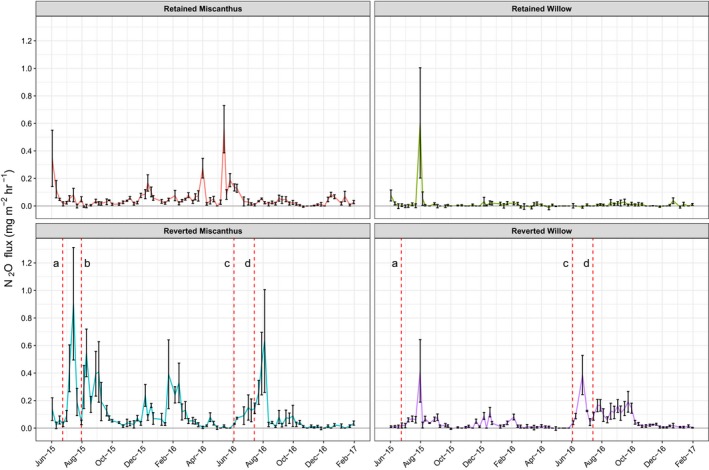
Time series N_2_O fluxes by land‐use, upper plots show retained controls while lower plots show reversion treatments. Values show the mean across two replicate plots (incorporating two subsample chambers within each replicate plot) with error bars representing the standard error. Dashed lines in reversion plots show the timing of intervention operations during the reversion process. (a) Initial spraying out and cutting back of both crops, (b) cultivation and seeding of forage kale (Miscanthus reversion plots only), (c) cultivation and seeding with ryegrass and (d) fertilisation of the new ryegrass plots (all reversion plots)

Examining the time series data (Figure 2) further demonstrates that both the magnitude and variability in N_2_O fluxes was generally lower in willow compared to Miscanthus, with the exception of a small number of time points. There was one particularly high flux in the retained willow, with a large standard error as this came from just one plot replicate; however, these high fluxes were consistent between subsample chambers within this plot so appeared to be a genuine flux rather than any measurement error (Figure 2). The mean flux rate across the entire study period for retained willow was extremely low at 0.015 mg ± 0.01 mg N_2_O m^−2^ hr^−1^. For the reverted Miscanthus the mean study period flux was 0.1 mg ± 0.02 mg N_2_O m^−2^ hr^−1^ peaking at 0.9 ± 0.4 mg N_2_O m^−2^ hr^−1^ on 15 July 2015. Table 1 provides a summary of mean and peak fluxes separately from pre‐ and postgrass sowing with ranges showing minimum and maximum flux values.

**Table 1:** Mean N_2_O fluxes sampled from both pre‐ and postgrass sowing with minimum and maximum values for each land‐use treatment

Crop		Retained		Reverted	
		Mean	Range	Mean	Range
Pre‐grass	Miscanthus	0.07 ± 0.01	−0.03 to 0.94	0.13 ± 0.02	−0.04 to 1.69
	Willow	0.02 ± 0.01	−0.06 to 1.69	0.03 ± 0.01	−0.04 to 0.84
Post‐grass	Miscanthus	0.03 ± 0.003	−0.02 to 0.19	0.08 ± 0.02	−0.03 to 1.64
	Willow	0.01 ± 0.001	−0.04 to 0.09	0.08 ± 0.01	−0.02 to 0.78

Note

Values given in mg N_2_O m^−2^ hr^−1^ (±*SE*).

### 3.2 Flux sums and GWP

The interpolated estimates for total emissions suggested the Miscanthus reversion plots had emitted more than twice as much N_2_O as the willow reversion plots: 3.41 ± 0.03 Mg CO_2_‐eq. ha^−1^ compared to 0.81 ± 0.01 Mg CO_2_‐eq. ha^−1^ during the study period. Total harvest offtake for the Miscanthus and willow over the entire cropping period (from the first harvest in 2010 to the last prior to reversion in 2015) was 82.41 ± 5.19 Mg DM ha^−1^ and 53.01 ± 1.59 Mg DM ha^−1^ respectively with estimated total energy produced over the cropping cycle being 1,479.26 ± 93.16 GJ ha^−1^ and 1,049.6 ± 31.48 GJ ha^−1^ for Miscanthus and willow respectively. Without including the reversion fluxes, the GWP cost of producing this energy would have been 6.50 ± 0.4 Mg CO_2_ eq. ha^−1^ for Miscanthus and 9.61 ± 0.3 Mg CO_2_ eq. ha^−1^ for willow. Adding on the reversion N_2_O costs increased the Miscanthus cost over the 6 year study period to 9.91 ± 0.4 Mg CO_2_ eq. ha^−1^ while for the willow this increased to 10.42 ± 0.3 Mg CO_2_ eq. ha^−1^. For Miscanthus particularly the reversion cost represented a significant increase, raising the carbon cost by 52%, for willow this was an increase of just 8.4%. The carbon cost of producing an equivalent amount of energy to each crop through coal would have been 178.82 ± 11.3 Mg CO_2_ eq. ha^−1^ for the Miscanthus and 126.88 ± 3.8 Mg CO_2_ eq. ha^−1^ for the willow. For Miscanthus this would be 18 times greater, for the willow this would be 12 times.

### 3.3 Regression modelling of the N_2_O flux measurements

Significant drivers retained following stepwise substitution differed markedly between the different land‐use treatments. These derived models, showing the retained parameters for each individual land‐use treatment, are presented below (see methods for full set of starting parameters used for all treatments).

***Retained Miscanthus…***

$$hboxN_{2}O∼T_{a}+\text{WFPS}\;+{\text{NH}}_{4}+T_{a}*\text{WFPS}+\text{WFPS}*{\text{NH}}_{4}+T_{a}*{\text{NH}}_{4}$$

***Reverted Miscanthus…***

$$hboxN_{2}O∼T_{s}+{\text{NH}}_{4}+{\text{NH}}_{4}/{\text{NO}}_{3}+{\text{NO}}_{3}+{\text{NH}}_{4}/{\text{NO}}_{3}*{\text{NO}}_{3}+{\text{NH}}_{4}*{\text{NO}}_{3}$$

***Retained willow…***

$$hboxN_{2}O∼{\text{NH}}_{4}+T_{s}+{\text{NO}}_{3}+\text{DOC}\;+\text{WFPS}+{\text{NH}}_{4}*{\text{NO}}_{3}+T_{s}*\text{WFPS}+T_{s}*{\text{NO}}_{3}+{\text{NH}}_{4}*\text{DOC}$$

***Reverted willow…***

$$hboxN_{2}O∼T_{s}+\text{WFPS}+\text{DOC}+{\text{NH}}_{4}+{\text{NO}}_{3}+{\text{NH}}_{4}*{\text{NO}}_{3}$$

*Asterisks in model structures depict interactions*.

The power of the derived regression models (adjusted *R* ^2^ to accommodate varying parameter numbers) to explain the variance in the individual N_2_O flux datasets differed notably between retained and reverted plots with models for the lower fluxes in the retained controls having less power. For retained Miscanthus total variance accounted for was 38.3%, for retained willow this was 46.0%. For the larger fluxes in the reversion treatments this increased to 44.9% for Miscanthus reversion and 63.2% for the willow reversion.

**Figure 3:**
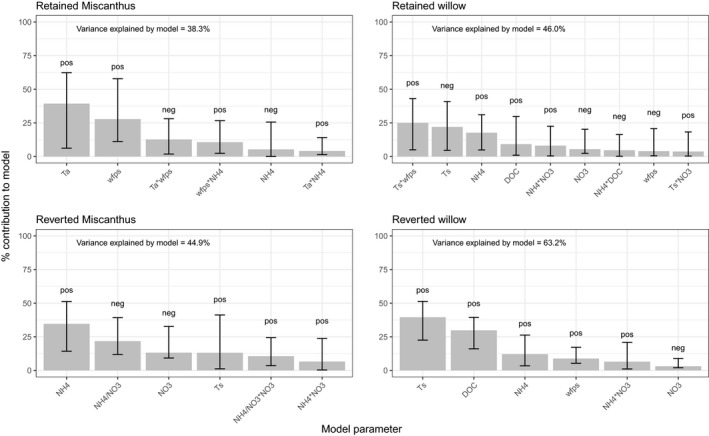
Relative importance of significant parameters within each regression model for individual land‐use treatments. Text within each plot shows how much of the total variance in N_2_O flux was explained by the corresponding regression model, the fits of all models were significant at *p *< 0.001. Bar heights (*Y* axes) show the percentage contribution that each parameter made to the total variance explained by each model. Parameters were excluded from the model if their inclusion showed no improvement to the model's AIC score. Error bars show bootstrapped estimations (100 samples) of 95% confidence intervals around each mean percentage contribution. Pos/Neg above each bar indicates direction of the influence of the parameter on the N_2_O flux magnitude

Figure 3 shows a graphical representation of the relative importance of these model parameters within each model. The relative importance of individual drivers, as well as the direction of their influence, varied between land‐use treatments. Temperature, whether soil (*T*_s_) or air (*T*_a_), and water‐filled pore space (WFPS) stood out as the primary drivers in the retained control plots. For retained Miscanthus two parameters (*T*_a_ and WFPS) provided 67.16% of the model's explanatory power with both parameters being positive drivers. For the retained willow it was *T*_s_ and its interaction with WFPS and NH_4_^+^ that appeared to be important, with these three parameters giving 64.75% of the model power and *T*_s_ appearing to be a negative driver in this model, that is N_2_O fluxes decreasing as soil temperature increased. The reverted plots in both treatments were quite different to their retained controls, particularly in the case of the Miscanthus reversion where drivers were dominated by nutrient status with NH_4_^+^, NO_3_^−^ and the interaction between them providing 69.61% of the model's power: increasing NH_4_^+^ and decreasing NO_3_^−^ contributed to higher N_2_O fluxes. Soil temperature was also a significant positive driver in this model, adding a further 13.13% to the model power. For the reverted willow plots *T*_s_ and labile carbon (DOC) became the most important drivers, providing 69.4% of the model's power between them, with both being positive drivers, though NH_4_^+^ was also important in this model, adding a further 12.11%.

### 3.4 Soil fertility

**Figure 4:**
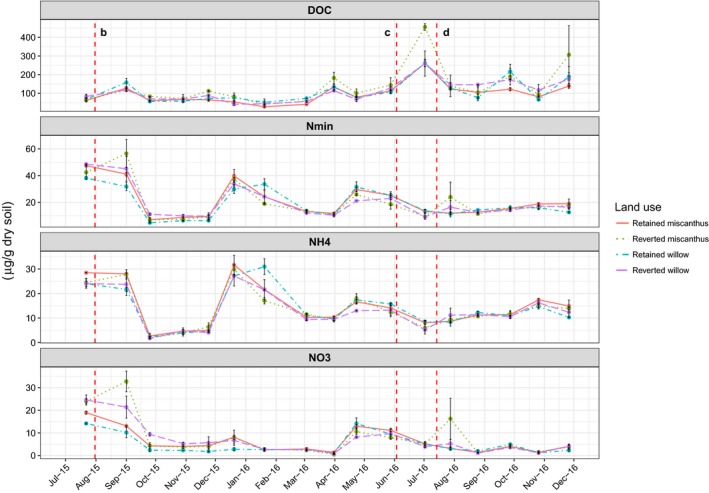
Monthly values (μg (g dry soil)^−1^) of total inorganic nitrogen (N_min_), ammonium (NH_4_) and nitrate (NO_3_) and dissolved organic carbon (DOC) in the 0–15 cm soil layer by crop and land‐use treatment across both years of the study. Error bars show *SE* of the mean of the two replicate plots per treatment, each replicate plot value is the mean of two subsamples. As in Figure 2, vertical dashed lines indicate the timing of soil interventions in the reverted plots, (b) cultivation and seeding of forage kale (Miscanthus reversion plots only), (c) cultivation and seeding with ryegrass (all reversion plots) and (d) fertilisation of the new ryegrass plots (all reversion plots)

Soil total inorganic N (N_min_) concentrations fluctuated over time with similar temporal trends in both retained and reverted treatments in both crops (Figure 4). However, some differences were apparent between treatments, with a rise in N_min_ found in the Miscanthus reversion plots seen in the September sampling, following cultivation and seeding with kale in August, all other plots showed a decrease at the same sampling. Total N_min_ in these plots reached a peak of 56.5 ± 10.5 μg N (g dry soil)^−1^ (63.6 ± 11.82 kg N ha^−1^). This was primarily driven by increased levels of NO_3_^−^ rather than NH_4_^+^ (Figure 4), this was reflected statistically with no significant differences between crops or treatments for total N_min_ or NH_4_^+^, however there were significant differences between retained and reverted treatments for NO_3_^−^ at this sampling with reverted plots being on average 0.9 μg NO_3_^−^ (g dry soil) ^−1^ higher (*p* = 0.02, *t* = 3.64, *df* = 4). A similar trend in the rise of N_min_ was seen again in the reverted Miscanthus at the end of July 2016. Again driven by NO_3_^−^, though at this time point the differences were not shown to be significant, though close to the threshold (*p* = 0.058, *t* = 2.63, *df* = 4).

DOC concentration in all treatments followed similar trends and magnitude with the exception of a small number of time points where concentrations were seen to be higher in the reverted Miscanthus. Figure 4 shows DOC peaking in this treatment at 455.5 ± 17.7 μg C (g soil)^−1^ at the beginning of July 2016 when the reverted Miscanthus was found to be significantly higher (*p* = 0.02, *t* = 3.71, *df* = 4) compared to the mean across the other treatments at the same time of 260.9 ± 76.8 μg C (g soil) ^−1^. Another spike in DOC was noted in these plots at the end of November 2016, however, differences between crops and treatments were not found to be significant.

### 3.5 Existing belowground biomass (BGB) in reversion plots prior to cultivation

Mean total BGB (roots and rhizomes combined) across both Miscanthus reversion plots was 2,101.4 ± 684.8 g ODM m^−2^, for rhizomes specifically this was 1,484.3 ± 286.5 g ODM m^−2^. For the subsequent crop, forage kale, there was a mean BGB of 71.8 ± 39.6 g ODM m^−2.^

For willow the mean BGB (roots and stools combined) across both plots was 1,504.2 ± 72.4 g ODM m^−2^, for stools specifically this was 1,001.7 ± 60.4 g ODM m^−2^.

Pooling BGB for each crop, stools/rhizomes and recovered roots, there were no significant differences in biomass found between the Miscanthus and willow (*p* = 0.61; *F* = 0.29; *df* = 1).

### 3.6 Carbon and Nitrogen in belowground biomass

#### 3.6.1 Carbon

There were significant differences found between the percentage carbon contents of the belowground biomass components (roots, rhizomes/stools) for each crop (*p* = <0.001, *F* = 21.43, *df* = 2) though not in all components. Tukey's HSD showed Miscanthus C percent in roots (41.7 ± 0.8%) to be not significantly different to kale (38.2 ± 1.5% (*p* = 0.06)) or willow (43.6 ± 0.2% (*p* = 0.5)) although willow roots were significantly higher in carbon than kale roots (*p* = 0.002). Willow stools (44.7 ± 0.3%) and Miscanthus rhizomes (45.7 ± 0.2%) were not significantly different to each other (*p* = 0.93) with willow roots and stools also containing statistically similar percentages of carbon (*p* = 0.93). In contrast, Miscanthus rhizomes had significantly higher carbon content than Miscanthus roots (*p* = 0.03).

#### 3.6.2 Nitrogen

Similar to the carbon contents, the percentages of nitrogen found in belowground biomass components were also significantly different between crops and specific components (*p* = 4.72 × 10^−7^, *F* = 44.804, *df* = 2). N percentage in Miscanthus roots (1.02 ± 0.07%) was not significantly different to kale roots (1.3 ± 0.1%), (*p* = 0.07) or willow roots (0.7 ± 0.1%), *p* = 0.07) though the willow roots were found to be significantly lower in nitrogen than the kale roots (*p* = 0.0002). Willow stools (0.4 ± 0.02%) were also significantly lower in percentage N than Miscanthus rhizomes (1.0 ± 0.1%), (*p* = 0.0002). For Miscanthus, roots and rhizomes were not significantly different to each other; in contrast, willow stools had significantly less percentage N than willow roots (*p* = 0.046).

#### 3.6.3 C/N ratios

C/N ratios for Miscanthus rhizome and willow stools were 47.1 and 128.1 respectively while for willow, Miscanthus and kale roots they were 64.5, 41.5 and 29.4.

Pooling all components and considering the C/N ratios of the total BGB added to the soil in each crop during the reversion process, there was a significant difference found between crops (*p* < 0.001, *F* = 13.6, *df* = 2). The belowground biomass in the willow reversion had a significantly higher C/N ratio than both the Miscanthus (*p* < 0.01) and the kale (*p* < 0.001). However, there was no difference found in the overall C/N ratios between the Miscanthus and the kale (*p* = 0.59).

### 3.7 C and N additions to soils in reversion plots

Averaged across plots, these percentages suggested that the first cultivation in the Miscanthus reversion process had added a total of at least 935.5 ± 139.5 g C/m^2^ (roots and rhizome combined) and 21.1 ± 3.1 g N/m^2^ to the soil. The following kale step in the Miscanthus reversion pathway added a further 36.1 ± 23.2 g C/m^2^ and 1.2 ± 0.8 g N/m^2^. For the single addition of roots/stools in the willow reversion, this added less C than the Miscanthus reversion at 666.7 ± 25.7 g C/m^2^ and less N at 7.03 ± 0.3 g N/m^2^.

### 3.8 Annual crop harvest yields, retained Miscanthus and willow control plots

Over the entire cropping period, 2010 to 2016, the Miscanthus yielded, on average, 1,373.5 ± 193 g ODM m^−2^ yr^−1^ (discounting the first year's growth in 2009). For the period directly relating to the present study, the 2015 and 2016 growing seasons, the retained control plots yielded a mean biomass of 1,376.8 ± 49.7 g ODM/m^2^ and 995.6 ± 32.7 g ODM/m^2^, respectively. As willow was harvested only every 2 years results are divided by two to give an annual estimate. This gave a mean annual yield for the willow of 884.5 ± 33.6 g ODM/m^2^ between 2010 and 2015 (again discounting the first year's growth). For 2015 specifically this was 990.3 ± 47.7 g ODM/m^2^.

### 3.9 Climatic data

**Figure 5:**
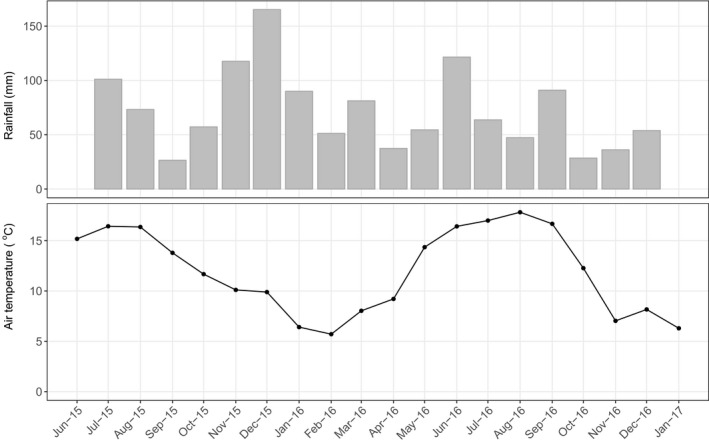
Monthly total rainfall (mm) and mean daytime air temperature (daytime defined as periods with incoming solar radiation >10 W/m^2^)

Figure 5 shows the variability in monthly rainfall and air temperature over the study period; winter 2015–2016 (November to February) was wetter and warmer (424 mm total rainfall, 8.03°C av. air temp.) than winter 2016‐2017 (231.9 mm and 6.9°C). The two growing seasons (June to October) also differed with 2015 being drier and cooler (301.9 mm and 14.7°C) than 2016 (352 mm and 16.03°C) though the difference in rainfall was driven primarily by a much wetter June in 2016 compared to 2015 (121.5 mm compared to 43.9 mm). For comparison, regional 30 year averages (1981‐2010) show a mean winter temperature at 7.2°C, and rainfall of 451.7 mm, while for the growing season, mean temperatures were 17.02°C with rainfall at 464.5 mm (Met office 2018).

## 4 DISCUSSION

### 4.1 N_2_O flux

The results showed that N_2_O fluxes were significantly increased by agronomic operations carried out during the reversion pathways in each of the crops. Not only did soil perturbation increase flux magnitude but, for the reverted Miscanthus plots particularly, it increased the variability between replicates markedly as well.

N_2_O fluxes in the retained willow plots were remarkably low, with far less activity than in the retained Miscanthus. Published N_2_O flux data for mature willow are not common but studies (Drewer et al., 2012, 2017; Gauder et al., 2012) give very low mean fluxes of −0.0003 mg to 0.0003 mg N_2_O m^−2^ hr^−1^ with peaks from 0.01 mg to 0.05 mg N_2_O m^−2^ hr^−1^. Figures for the retained willow in this study were somewhat higher than these means but still extremely low compared to the other treatment plots with a mean flux rate across the entire study period of 0.015 mg N_2_O m^−2^ hr^−1^. One study, (Kavdir, Hellebrand, & Kern, 2008) did report higher peak fluxes in unfertilised willow at 0.21 mg N_2_O m^−2^ hr^−1^ though their mean flux rate remained low at 0.01 mg N_2_O m^−2^ hr^−1^, directly comparable to our present study despite being carried out on lighter, sandy, soils.

There was little difference between retained and reverted willow plots in the pregrass period, showing that merely killing off and cutting back the original crop had little effect on soil N_2_O fluxes, it was not until these reverted plots were subsequently cultivated and sown with the ryegrass crop and fertilised that N_2_O flux activity increased significantly. From this point on there was no significant difference between the fluxes from either crop reversion plots, with flux magnitudes at the lower end of literature ranges for grassland (see below). This suggests that the N_2_O impacts of reversion were largely contained within the reversion period itself with fluxes from the cultivation activities for the following crop dominating any flux legacy from the previous energy crops.

For the Miscanthus plots, the reversion pathway undertaken appeared to have a much higher impact on N_2_O flux with both magnitude and variability increased significantly. Fluxes decreased notably into the postgrass period but remained higher than the retained controls. This suggested that the reversion process for the Miscanthus increased N_2_O flux more than the subsequent cultivation to ryegrass and its fertilisation, where fluxes decreased and became not significantly different to the reverted willow plots. Literature studies for Miscanthus tend to concentrate on land‐use transitions and fertiliser manipulations though there are figures from experimental controls available for mature unfertilised Miscanthus crops (Behnke et al., 2012; Drewer et al., 2012; Gauder et al., 2012; Jørgensen et al., 1997; Roth et al., 2013, 2015), mean fluxes range from ‐0.002 mg to 0.157 mg N_2_O m^−2^ hr^−1^ with peak fluxes from 0.006 mg to 0.7 mg N_2_O m^−2^ hr^−1^. Results from the retained Miscanthus controls in our study are well within this with a mean of 0.054 mg N_2_O m^−2^ hr^−1^ and a peak of 0.56 mg N_2_O m^−2^ hr^−1^.

### 4.2 Drivers of N_2_O emission

The significance and relative importance of some of the known drivers of N_2_O flux in soils were investigated statistically using multiple regression between fluxes and measured parameters (Clayton, McTaggart, Parker, Swan, & Smith, 1997; Moulin et al., 2014; Roth et al., 2013; Shepherd, Barzetti, & Hastie, 1991). It is particularly interesting to note that while soil nitrogen and labile carbon levels were not often significantly different between the treatments, the modelled responses to them varied notably between the both the treatments and crops. Results highlighted differences in the drivers between the land‐uses suggesting that flux dynamics could not always be explained by simple single metrics such as soil temperature and water‐filled pore space (WFPS). For the retained *Miscanthus*, the primary positive drivers were air temperature and WFPS, reflecting the general consensus as to the importance of these factors; that is warmer wetter conditions produced more N_2_O. In contrast though, for the reverted Miscanthus plots, the nitrogen status and in particular the NH_4_^+^ to NO_3_^−^ ratio, appeared to be stronger determinants. The modelling suggested that as NH_4_^+^ increased and NO_3_^−^ decreased the N_2_O flux increased, perhaps suggesting denitrification of NO_3_^−^ due to oxygen utilised in the immobilisation of the available NH_4_^+^ by soil bacteria.

For the low fluxes of the retained willow all drivers seemed to be playing a role which might suggest some over‐fitment of the model in this treatment, though again, soil moisture and NH_4_^+^ status were the main drivers. In this case, perhaps counterintuitively, soil temperature was a negative driver, that is as soils warmed N_2_O production decreased. This may be difficult to explain with the data available but it should be noted that fluxes in this treatment were generally extremely low. For the higher fluxes in the reverted willow soil, explanation of variance was the highest of the four land‐use treatment models; soil temperature and labile carbon stood out as the main drivers closely followed by NH_4_^+^ and WFPS, all positive influences. This might suggest the main process being captured here was the decomposition of organic carbon from the high C/N ratio willow stools taking up oxygen from the soil leading to oxygen limitation and denitrification of soil NO_3_^−^. This suggestion might be supported by the negative influence of NO_3_^−^ in this model, that is as NO_3_^−^ decreased N_2_O flux increased, though it must be noted the NO_3_^−^ concentrations in this model were only playing a weak part in the explanatory power.

### 4.3 GHG cost of biomass production and reversion

Plots for both reverted crops exhibited significantly higher mean and peak flux rates in our study than their retained controls, demonstrating that there is a GHG penalty to be paid when these crops are returned to grassland. These GHG costs were significantly higher for the Miscanthus reversion pathway than for the willow in the pregrass period. The regression modelling suggested that this was largely driven by the increased soil disturbance and higher nitrogen contents of the biomass incorporated into the soils during this period in the spraying/cultivation/forage kale pathway when compared to the spraying/fallow treatment in the willow plots. However, it should be noted that while there are N_2_O costs associated with these reversion processes that need to be accounted for in crop cycle GHG balance estimations, they do need to be considered against the typical emissions of alternative cropping systems. Arable systems have been demonstrated to reach peak N_2_O emission rates between 0.02 to 1.75 mg N_2_O m^−2^ hr^−1^ (Drewer et al., 2012, 2017; Gauder et al., 2012; Jørgensen et al., 1997; Parkin & Kaspar, 2006), potentially double the highest Miscanthus reversion fluxes. Routine grassland N_2_O emission peaks can be orders of magnitude greater than the highest short‐term energy crop reversion fluxes reported here, with peak figures for grassland in the literature, dependant on management, ranging from 0.18 to 12.43 mg N_2_O m^−2^ hr^−1^ (Clayton, Arah, & Smith, 1994; Flechard et al., 2007; Jones et al., 2011; Rafique, Hennessy, & Kiely, 2011; Yamulki & Jarvis, 2002). Our postgrass peak figures at 0.78 mg N_2_O m^−2^ hr^−1^ (willow) and 1.64 mg N_2_O m^−2^ hr^−1^ (Miscanthus) are at the lower end of this range and could be regarded as typical grassland fluxes.

In terms of the GWP (CO_2_ eq.) cost of the reversion fluxes, there is little doubt that reversion added a significant cost to the overall energy production, particularly in the Miscanthus. This study, though, only considered the energy production over a 6 year crop lifetime, it would be more typical to expect a 15 or even 20 year lifespan for these perennial crops, the single year reversion cost would then be a much smaller proportion of the overall energy production. Furthermore, the LCA study (Hastings et al., 2017) which produced the CO_2_ eq. cost of biomass production did not consider the potential for soil carbon changes under these crops as this is extremely site and soil specific. It might be expected that potential soil carbon gains or losses could significantly change the CO_2_ eq. costs of production (either positively or negatively), again impacting the overall percentage that reversion fluxes play in the total cost of production.

## 5 CONCLUSION

This study represents the first investigation of N_2_O fluxes following the end of energy crop cycles. In order to achieve a side‐by‐side comparison of willow and Miscanthus reversion we utilised a pre‐existing experiment established in 2009. Despite being restricted to two replicates plots in each treatment the increase in the observed N_2_O flux following reversion was unequivocal for our experiment. Our results, in the context of a simple LCA, suggest that real GHG savings can still be achieved relative to fossil fuel usage. Given the current paucity of data relevant to our study we propose that further work is required to understand reversion impacts for a variety of feedstocks, soil types and climates, ideally incorporating high frequency sampling for nutrient dynamics and soil microbial activity. New work should also include data arising from real‐world commercial reversion and with consideration to changes in soil organic carbon which can be a major determinant of the longer‐term GHG balance. Herein lies a significant challenge in identifying locations with detailed land‐use history and with adjacent reliable counterfactual paring. A final consideration we raise, from a policy perspective, is whether it will be appropriate to allocate both conversion and reversion GHG emissions to the cultivation of the bioenergy crop.
